# Beta Cell Imaging—From Pre-Clinical Validation to First in Man Testing

**DOI:** 10.3390/ijms21197274

**Published:** 2020-10-01

**Authors:** Stephane Demine, Michael L. Schulte, Paul R. Territo, Decio L. Eizirik

**Affiliations:** 1 Indiana Biosciences Research Institute, Indianapolis, IN 46202, USA; deizirik@ulb.ac.be; 2 Department of Radiology and Imaging Sciences, Indiana University School of Medicine, Indianapolis, IN 46202, USA; schultml@iu.edu (M.L.S.); pterrito@iupui.edu (P.R.T.); 3 Division of Clinical Pharmacology, Department of Medicine, Indiana University School of Medicine, Indianapolis, IN 46202, USA; 4 ULB Center for Diabetes Research, Medical Faculty, Université Libre de Bruxelles (ULB), 1070 Brussels, Belgium

**Keywords:** beta cell imaging, MRI, pancreas, PET, pre-clinical validation, radiochemistry, SPECT, type 1 diabetes, type 2 diabetes

## Abstract

There are presently no reliable ways to quantify human pancreatic beta cell mass (BCM) in vivo, which prevents an accurate understanding of the progressive beta cell loss in diabetes or following islet transplantation. Furthermore, the lack of beta cell imaging hampers the evaluation of the impact of new drugs aiming to prevent beta cell loss or to restore BCM in diabetes. We presently discuss the potential value of BCM determination as a cornerstone for individualized therapies in diabetes, describe the presently available probes for human BCM evaluation, and discuss our approach for the discovery of novel beta cell biomarkers, based on the determination of specific splice variants present in human beta cells. This has already led to the identification of DPP6 and FXYD2γa as two promising targets for human BCM imaging, and is followed by a discussion of potential safety issues, the role for radiochemistry in the improvement of BCM imaging, and concludes with an overview of the different steps from pre-clinical validation to a first-in-man trial for novel tracers.

## 1. Beta Cell Mass Evolution during Type 1 and Type 2 Diabetes

Diabetes currently affects 420 million people worldwide, and this prevalence is projected to increase to 642 million people by 2040 [1]. The disease is associated with high morbidity, causing five million deaths annually worldwide [1]. Diabetes-related deaths are primarily due to cardiovascular diseases (50%) and kidney failure (10–20%) [2,3,4]. Diabetes is also the leading cause of blindness in working adults and contributes to 50% of lower limb amputations [5]. Type 1 diabetes (T1D, around 10% of the cases) is caused by a progressive and eventually nearly complete loss of beta cell mass (BCM) following autoimmune destruction [6,7]. In contrast, type 2 diabetes (T2D, 85% of the cases) is caused by a failure of pancreatic beta cells to compensate for insulin resistance in peripheral tissues [8]. This form of the disease is typically associated with obesity and leads to progressive beta cell failure due at least in part to chronic metabolic stress [8]. The clinical diagnosis of diabetes relies on the detection of elevated blood glucose levels or the surrogate marker HbA1C (glycated hemoglobin) [9]. T1D patients are treated by multiple daily injections of insulin to control blood glucose levels. In some cases, i.e., recipients of a kidney graft or patients affected by recurrent and severe hypoglycemia, islet transplantation is an option, allowing partial recovery of endogenous insulin secretion and, in most cases, transitory independence from exogenous insulin administration [10,11]. The initial therapies for T2D rely on lifestyle changes (weight loss and exercise) and the use of drugs stimulating the release of endogenous insulin and/or increasing the peripheral sensitivity to this hormone, with the eventual addition of exogenous insulin [9].

Both T1D and T2D are characterized by a reduced BCM [6,7,8,12,13], but the natural history of beta cell loss in these diseases, as well as the relationship between BCM and function, remains unclear. This is due to a lack of adequate tools for the in vivo quantification of human BCM. Currently, most studies are limited to pancreatic specimens from necropsy. Given the medical complications and ethical restrictions associated with pancreatic biopsy, this method is not a viable approach for measuring BCM. In T1D, the autoimmune attack against pancreatic beta cells results in progressive and nearly complete loss of these cells [14,15,16]. However, many studies reported the existence of remaining beta cells and detectable C-peptide in the circulation in >30–80% of long-duration T1D patients [17,18,19,20]. In T2D patients, the decrease in BCM is more modest (by 22–63%) [21,22]. In both forms of diabetes, BCM loss is preceded by progressive loss of beta cell function, as was confirmed by studies performed on pancreas slices from T1D organ donors [23]. It is thought that many beta cells are preserved as quiescent or “stunned” cells (i.e., degranulated or not releasing insulin) in both T2D [24] and T1D [17,18,25]. This represents a challenge for histological determination of BCM in tissue specimens as beta cells may still be present but missed when anti-insulin staining is used. Therefore, BCM is probably often underestimated [26,27].

The beta cells that may still be viable in diabetic patients are, therefore, good targets for future pharmaceutical interventions. How these residual beta cells influence the course of the disease is currently unknown. Do they help stabilize glycemia, reduce the rate of diabetes complications, or prevent severe episodes of hypoglycemia? It is crucial to underline that clinical diabetes depends not only on the absolute amount of beta cells but also on the function of the individual beta cells. Patients with diabetes or impaired glucose tolerance tend to have a lower BCM, but the BCM by itself does not necessarily predict the glucose tolerance of individual patients. Whether knowledge of BCM in relation to beta cell function helps to predict if and when an individual will develop diabetes, and who will respond or not to certain therapies, remain to be defined [28].

## 2. Beta Cell Imaging Is a Cornerstone for Future Individualized Treatment of Diabetes

The great Brazilian poet Mario Quintana once remarked, *“The correct answer is irrelevant: the essential is that the questions are correct”.* However, to reach successful human beta cell imaging, we will need both correct questions and correct answers. The best present approach to quantify BCM is medical imaging. This technique is non-invasive, fast, safe, quantitative, and can be used repeatedly in the same patients. Medical imaging machines are also widely available. Beta cell imaging would be ideal at patient diagnostics to identify the best-suited therapeutic strategies based on the remaining BCM, to ensure the patient’s follow-up, and to assess their responses to novel therapies aiming to prevent beta cell loss or to restore BCM. For example, it would help to identify those individuals with T2D that would benefit from therapies relying on the presence of a large amount of viable, potentially insulin-secreting beta cells, such as sulfonylureas or GLP-1 (glucagon-like peptide-1) analogs, while others with very limited beta cell reserve may directly change to insulin replacement. In the case of T1D, the presence of a good reserve of non-functional beta cells may indicate the use of anti-inflammatory agents (e.g., cytokine blockers) in parallel to insulin therapy, with the hope of restoring some endogenous insulin release [29]. BCM imaging could also be used to assess the survival of islets or pancreas grafts and to guide the selection of immunosuppressive treatments to reduce graft rejection. Beta cell imaging would also be crucial to enhance our understanding of the pathophysiology and disease progression of both T1D and T2D. Finally, beta cell imaging could be an invaluable tool for drug development, used for the validation of new therapeutic compounds aiming to restore BCM and function. By helping in the stratification of patient cohorts, it would help to reduce costs, improve clinical trial reliability, and reduce the clinical trial attrition rate. Ideally, these methods should be used in parallel of C-peptide determination, which would allow the detection of both functional beta cells (beta cell mass and stimulated C-peptide are in agreement) and non-functional beta cells (beta cells are present, but there is no or very low stimulated C-peptide).

Despite this clear potential, the ideal beta cell-specific imaging probe has yet to be identified. This can be explained by the many obstacles hampering the development of such techniques. One of the major obstacles is that beta cells constitute only 1–3% of the total pancreatic mass and are heterogeneously distributed throughout the pancreas into the small islets of Langerhans (100–300 µm in diameter) [30]. Islets themselves are composed of multiple cell types, including beta (~60%), alpha (~30%), delta (~10%), PP (pancreatic polypeptide), epsilon, endothelial, and neuronal cells [30]. There are also marked inter-individual differences in BCM independently of disease [13,22,31], and BCM mass in people with T2D has substantial overlap with BCM of non-diabetic individuals and patients with impaired glucose tolerance [32]. Finally, beta cell dysfunction(s) and the pro-inflammatory environment in T1D or the metabolic stress in T2D lead to considerable changes in gene expression profile [14,33,34,35,36], which complicates the identification of a biomarker suitable for beta cell quantification across disease states. Therefore, the ideal probe/target should be exquisitely beta cell-specific and sensitive enough to allow discrimination between healthy individuals and diabetic patients without being affected by beta cell stress secondary to disease pathogenesis.

Currently, attempts at in vivo visualization of beta cells in humans rely on radiolabeled tracer molecules that bind to beta cells with different degrees of specificity [37]. These radiotracers can be detected at the picomolar range by two techniques: positron emission tomography (PET) and single-photon emission computed tomography (SPECT) (see details below, in part 8.). Although the spatial resolution of both types of scanners does not allow resolving single islets [38], beta cell quantification by imaging actually does not require the resolution of single islets. Indeed, the “visualization” of beta cells is based on the high specificity and the biochemical/metabolic characteristics of the tracer molecule (chemical resolution) [37,39] that provides an estimation of the total beta cell mass. These techniques must be used in conjunction with anatomical imaging techniques such as magnetic resonance imaging (MRI) or computed tomography (CT) [38], which allows organ segmentation, an useful method to ascertain the origin of the PET or SPECT signal. For BCM imaging, PET is preferred over SPECT as both the resolution and the sensitivity of clinical PET imagers are higher than SPECT instruments (4–5 mm and >10 mm, respectively) [38]. MRI alone has also been used for BCM quantification and visualization. While MRI exhibits excellent resolution (<0.5 mm), this degree of resolution is not widely available clinically [38]. Furthermore, this method suffers from lack of sensitivity and requires the accumulation of probes in the micromolar range [40], which currently limits its use for BCM imaging. In light of the continuous technology improvement in the field [41], it is conceivable that MRI-based technologies for visualization of islets in humans will become widely available in the coming years.

## 3. BCM Probes Presently under Clinical Investigation

During the last decade, several putative beta cell-specific probes have been identified, including monoclonal antibodies (mAbs) [42,43], short or fragments of antibodies [44,45], peptides [46,47,48], small molecules such as metabolites [49,50,51], and ion-binding probes [52,53]. The different existing tracers have been recently and comprehensively reviewed [54] and will not be further discussed here. Amongst this variety of probes, only three families of radiotracers are currently undergoing clinical evaluation in humans: ^18^F-dihydro-tetrabenazine (DTBZ) for imaging of vesicular monoamine transporter 2 (VMAT2) [49,55], ^11^C-hydroxytryptophan (HTP) as a marker for the serotonergic system [50], and ^111^In- and ^68^Ga-labeled exendin-4 and derivatives targeting the GLP-1 receptor [46].

Exendin-4 and derived molecules are presently the most promising approach for BCM imaging. Many studies performed in rodents found a tight correlation between BCM and exendin-4-associated signal [56,57]. This probe is highly sensitive and can be used to detect as few as 50 rat islets transplanted intramuscularly in mice [58]. Exendin-4 can also be used to monitor endogenous BCM evolution in mice after drug intervention. Thus, after nine weeks of canagliflozin treatment (a sodium-glucose transporter-2 inhibitor), an increase in BCM and accumulation of [Lys^12^(^111^In-Bn-diethylenetriaminepenta-acetic acid(DTPA)-Ahx)]exendin-4 was observed in mice [59]. The same probe was also used to assess the protective effect of DS-8500a (a novel G protein-coupled receptor-119 agonist) on BCM in *db*/*db* mice, a model of severe insulin resistance and diabetes [60]. Importantly, insulitis does not affect the binding of [^111^In]In-DTPA-exendin-3 in NOD (non-obese diabetic) mice [61], but high glucose leads to decreased GLP-1 expression and consequent exendin binding [62]. Exendin-4-based probes are often used in conjugation with SPECT or PET but attempts to generate MRI probes have also been made. Recently, exendin-4 was conjugated to dodecane tetraacetic acid (DOTA) and conjugated with gadolinium (Gd) and was used to discriminate between healthy and β-cell-depleted C57BL/6J mice [63]. Altogether, these data suggest that exendin-4 is sufficiently specific and sensitive to be used for longitudinal BCM quantification in rodents, including during early diabetes. However, despite these promising results, studies performed in humans indicate some limitations. Thus, there was only a decrease by 40–60% of the exendin-derived signal in long-term T1D patients [56], which are expected to show a >90–95% decrease in BCM [64]. Possible explanations for this are: 1. GLP-1R expression may not be sufficiently beta cell-specific in humans. Indeed, this receptor was found expressed in alpha cells [65] and recent studies using super-resolution microscopy and fluorescent exendin-4 derivatives confirmed that GLP-1R is present in at least 5% of alpha cell population [66]. Low levels of expression were also found in other pancreas-surrounding tissues such as the intestine, stomach, lung, and kidneys [67,68], which could increase the background signal; 2. Another intriguing possibility is that exendin-4 detects non-functional beta cells, but this remains to be proven.

A similar lack of specificity was observed with the other probes currently under clinical investigation. Only 30% of the ^18^F-DTBZ signal originated from beta cells, the remaining 70% coming from the other pancreatic endocrine cells [69,70]. Another study found a moderate signal decrease (+/−50%) in type 1 diabetic patients using [^18^F]FP-(+)-DTBZ and PET imaging [55,71], while no difference was observed for type 2 diabetic patients [55]. A substantial and highly significant reduction in pancreas uptake was found in type 1 diabetic patients imaged with [^11^C]-HTP [50], but conflicting studies reported that HTP also accumulates in other pancreatic endocrine or exocrine cells [72]. Even if these probes may not be specific enough to quantify BCM with sufficient accuracy, they could be repurposed for the detection of insulinomas or the follow-up of islets grafts. In line with this, a recent study proved the efficiency of exendin-4 to detect insulinomas [73].

In conclusion, and in spite of the promising results with the tracers described above, there remains a clear need for the discovery of novel and more specific biomarkers to improve the potential for radiotracer based BCM imaging.

## 4. Detection of Transplanted Human Islets by MRI

As mentioned above, islet transplantation is a possible therapy for type 1 diabetes, particularly in patients affected by severe and recurrent hypoglycemia. Since the publication of the Edmonton protocol for immunosuppression, the success rate of islet transplantation in humans has improved [74]. A long-term endogenous insulin production and glycemic stability can be achieved in many patients, although insulin independence is progressively lost in spite of continuous immunosuppression [10,11,75,76]. Graft survival and function are usually assessed by measuring circulating C-peptide, glycemia and glycated hemoglobin, but imaging could provide additional and valuable information on the remaining beta cell graft mass. Techniques such as bioluminescence or fluorescence imaging have been developed in rodents [77,78,79], but are difficult to translate to humans. Promising results have been obtained with the use of MRI to detect islets prelabeled with paramagnetic metals. For instance, MRI was used to quantify iron oxide-labeled allogeneic islets grafted under the kidney capsule or injected in the portal vein of baboons [80]. Superparamagnetic beads were also used to label islets and to successfully monitor the grafts in rodents [81,82,83]. Paramagnetic beads can also be conjugated with other molecules to form bifunctional probes. For instance, human islets were pre-loaded with beads conjugated to a small interfering RNA directed against caspase 3 and transplanted in baboons, which helped to maximize graft viability and to minimize the number of donor islets required [84]. There are concerns that the use of iron will contribute to catalyze the generation of oxygen free radicals, but available data suggest that human islet pre-labelling do not affect islet cell viability or beta cell function [85], although it requires a prolonged culture time which may increase the risk of infection. An alternative approach would be to use paramagnetic beads conjugated to antibodies. For instance, immunomagnetic beads coated with an antibody targeting the rat MHC class I antigen was used with success to image rodent islets grafted in rat liver [85].

Another approach involves encapsulating human islets into pre-labeled biocompatible capsules. For instance, iron oxide nanoparticles encapsulated within alginate hydrogel capsules containing viable islets were developed and successfully used in mice [86]. These capsules can be retrieved using a magnetic field and a dedicated retrieval device. MRI imaging can also be used to detect periportal steatosis, a possible complication of intraportal islet transplantation [87]. A concern regarding the techniques described above is the relative low stability of these beads in vivo. For instance, in humans prelabeled islets were detected by MRI only up to 24 weeks [88]. Therefore, this technique is better suitable for the short-term detection of grafted human islets: other imaging techniques are needed for the long-term follow-up of recipients of islet allografts. Probes presented in part 3 could be used for this purpose.

## 5. New Experimental Approaches to Identify Beta Cell Biomarkers

Many of the biomarkers identified so far originated from high-throughput analyses, such as RNA sequencing or proteomics. Genes/proteins preferentially expressed in beta cells are selected and validated in vitro and in vivo. Of note, most studies up to now focused on whole gene expression, which limits the number of possible hits. Alternative splicing is an mRNA processing mechanism that leads to the generation of multiple transcripts from a single gene [89,90,91]. As this process is cell type-dependent [89], many transcripts are likely to be expressed in beta cells only.

Another possible drawback is related to the way beta cell-specific probes are usually designed and tested. First, the probes are developed and validated in vitro by using binding and competition studies performed on cells overexpressing the gene of interest or directly on the targeted cells. Second, the probes are functionalized for imaging, labeled, and used to image endogenous rodent BCM or rodent islets grafted in rodent models. In case of success, the probes are then tested in human cells. This approach has, however, important limitations. There are many differences between species, and genes are often differentially expressed in mice and humans [92], including in beta cells [93]. Even in the case where genes are similarly expressed in both rodents and humans, the protein homology, including post-translational changes, is often limited. For the beta cell-specific probes based on metabolites, the pathophysiological differences existing between humans and other species are also a limiting factor [94,95,96,97]. Therefore, a robust biomarker in rodents may not be specific in humans. Probes designed against mouse proteins are also likely to present a lower affinity for the human protein due to weak homology. As molecular imaging is often based on the use of small molecules, the impact of amino acid differences across species is theoretically higher [98].

**Figure 1:**
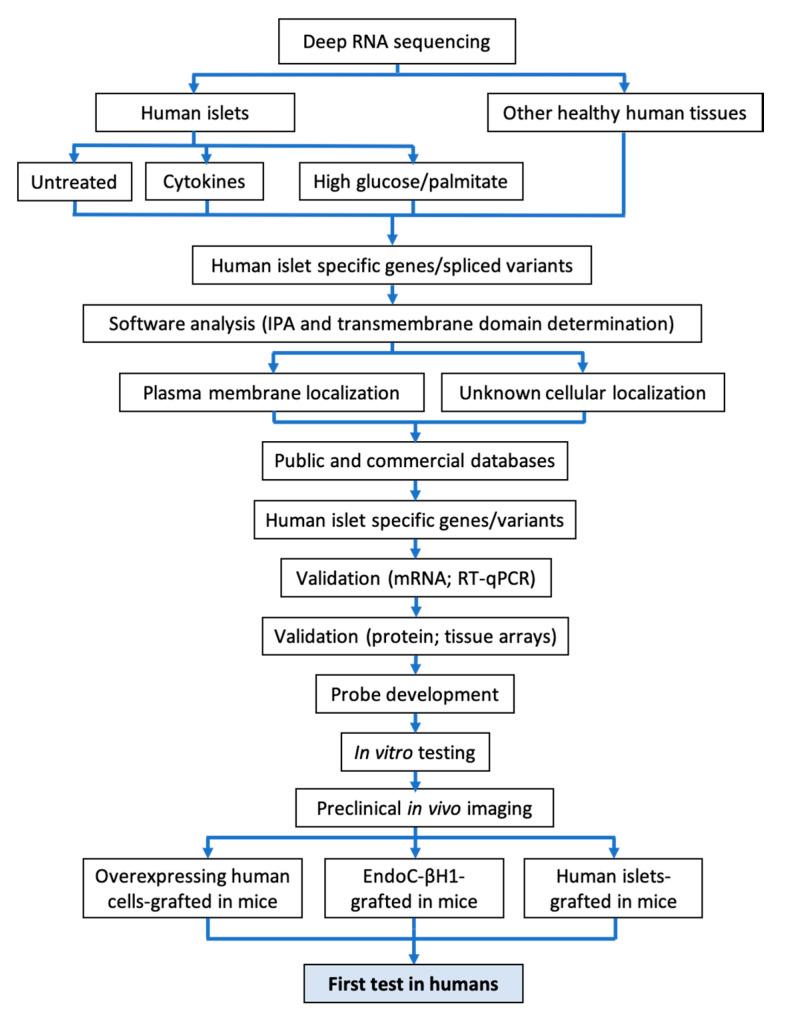
Step-by-step workflow presently used by our group to identify new beta cell biomarkers and to generate corresponding imaging probes. A schematic overview of the methodology used to mine RNA sequencing data for discovery of novel pancreatic islet biomarkers is shown. Transcripts differentially expressed in pancreatic islets are identified by comparing transcriptomes of human pancreatic islets against 16 different normal human tissues. Deep RNA sequencing analysis (>180 million reads, to allow identification of >80% splice variants) is performed on untreated human islet preparations or after treatment with pro-inflammatory cytokines (IL-1β + IFN-γ or IFN-α) or exposure to metabolic stress (high glucose and/or palmitate) to identify transcripts unaltered by the stressful conditions prevailing in diabetes. A software analysis (Ingenuity pathway analysis (IPA) analysis + transmembrane domain) is conducted on the generated hits to identify membrane-expressed proteins, potentially reachable with a probe. Once the biomarkers identified are validated at the mRNA and protein levels, one or more imaging probes are developed. After complete in vitro validation, the probes are tested for in vivo imaging on humanize mouse models grafted with different amounts of human beta cells.

Our group has approached the discovery of novel beta cell biomarkers by combining two proposed complementary approaches, namely, using human islets or beta cells from the start and focusing on the identification of specific splice variants present in human beta cells, but not in other human tissues [34,35,45,99,100]. Our present approach for biomarker discovery is shown in Figure 1. Only the plasma membrane proteins (or those with an unknown cellular location) that are not modified by exposure to the proinflammatory cytokines IL-1β, IFN-γ and IFN-α, or by metabolic stress (i.e., exposure to high glucose and/or saturated free fatty acids as palmitate), are considered for further validation. The rationale for this is that islets are probably exposed to these stresses during the course of diabetes [8] and we wish to avoid stress-induced changes in the expression of our biomarkers. So far, we identified two biomarkers by using this or a similar approach: dipeptidyl peptidase 6 (DPP6) [45] and FXYD domain-containing ion transport regulator 2γ (FXYD2γ) variant a [47,48]. We have developed imaging probes for each of them, based on small camelid antibodies (nanobodies, Nb) [45,101] or small proteins generated by phage display [47], respectively.

The *DPP6* gene encodes for a transmembrane protein of 98 kDa characterized by a short intracellular tail and a massive extracellular domain of 728 amino acids [102], which makes it a good candidate for targeting with imaging probes. In neurons, DPP6 acts as a regulatory subunit of the voltage-gated A-type Kv4.2 potassium channel complex, helping to stabilize it [103]. The role of this protein in beta cells remains to be determined. DPP6 autoantibodies were found associated with encephalitis and neurologic disorders in humans [104]. We have shown that DPP6 expression is restricted to beta cells, with a mild expression in alpha cells and a low level of expression in neurons, and that decreased DPP6 expression is present in long-term type 1 diabetic patients [45,101]. RNA sequencing data suggests that DPP6 expression is higher and better correlated to the expression of insulin than the GLP-1 receptor [45,101]. Of note, DPP6 is also highly expressed in insulinomas [45] and may become a novel biomarker for detecting these difficult-to-diagnose tumors.

**Figure 2:**
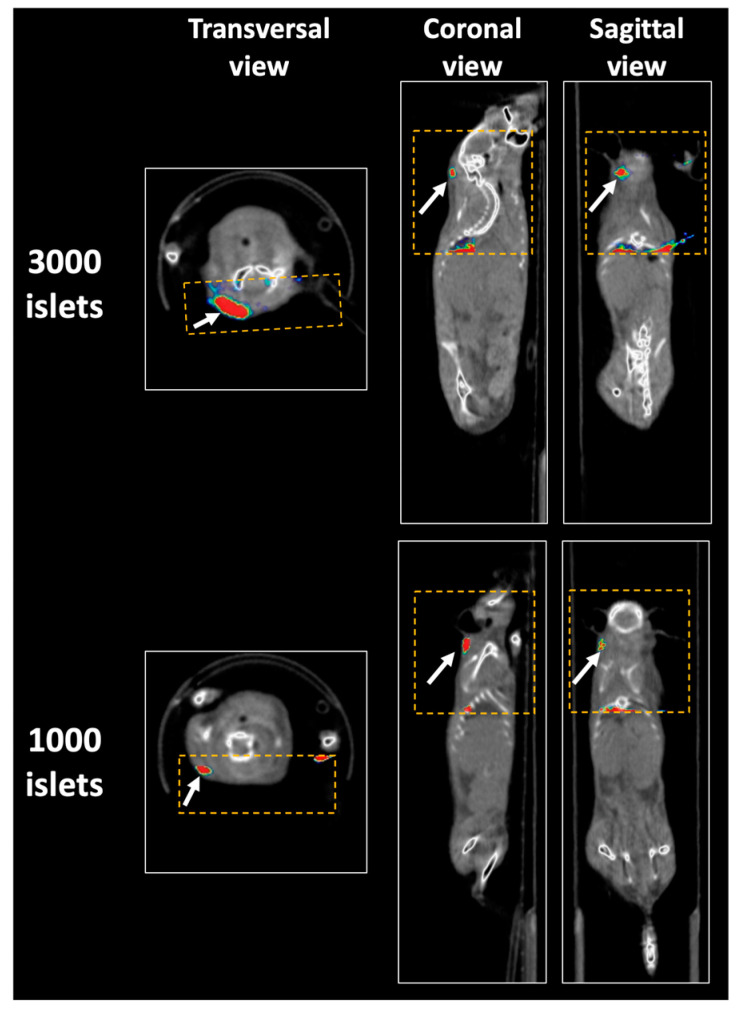
SPECT imaging of human islets grafted in immunodeficient mice using an anti-DPP6 nanobody. Female NOD-SCID mice were transplanted subcutaneously in the intrascapular area with different numbers of primary human islets (1000 or 3000 islet equivalent (IEQ)) as described [101]. After 4 weeks, the mice were imaged 60 min post-injection by full body CT followed by a focal single-photon emission computed tomography (SPECT) imaging scan using an ^99m^Tc-anti-DPP6 nanobody. The yellow square indicates the field-of-view of the SPECT camera. The white arrows indicate the graft localization. Full analyses and quantification of these pictures are described in [101].

To develop an anti-DPP6 probe, we used an innovative approach based on a camelid antibody, named nanobodies (Nb) [45]. These small (10–14 kDa) antibodies display unique features with respect to size, stability, and versatility [105,106]. They present thus advantages over the 10-times larger traditional antibodies. For instance, based on their unique pharmacokinetics it is possible to image mice 60 min post Nb injection [45], while imaging with mAbs is ideally performed 2–4 days post administration [107]. This timing also allows the use of radioisotopes with a shorter half-life. With an apparent biological half-life estimated to 3–4 h [108], most of the Nb is quickly cleared from the patient’s body via the urinary system, avoiding prolonged exposure to radioactive isotopes or possible probe-associated cytotoxic effects. By using SPECT and an anti-DPP6 probe, we successfully imaged mice grafted with tumors formed by cells overexpressing the protein of interest [45]; human clonal beta cells (EndoC-βH1) [45]; and human islets [101] (Figure 2). Furthermore, dose-response experiments using immune-deficient mice grafted with different numbers of EndoC-βH1 and human islets indicated a significant positive correlation between the probe uptake and the number of cells grafted [101]. However, due to the limited sensitivity of SPECT imaging, only the largest number of transplanted cells (>5 × 10^6^ cells or >3000 islet equivalents) provided a significantly higher signal as compared to the background signal observed following grafts of exocrine pancreatic tissue (which may still contain a small amount of islet cells) [101]. Future efforts will aim to adapt the anti-DPP6 Nb to PET imaging to improve sensitivity and quantitation. A first experiment, performed on NOD-SCID (Nonobese diabetic/severe combined immunodeficiency) mice grafted with human neuroblastoma cells naturally expressing DPP6 and imaged with ^68^Ga-radiolabeled anti-DPP6 Nb, confirmed better performance as compared to the previous version of the probe [101].

The second biomarker identified belongs to the FXYD protein family, which is composed of single transmembrane proteins containing an FXYD motif at their N-terminus [109]. The gene encoding FXYD2γ can produce two splice variants: FXYD2γa and FXYD2γb that only differ by their first exon (8 amino acids) encoding the extracellular N-terminal extremity of the protein [109]. FXYD2γa is predominantly expressed in kidney tubule cells, but limited expression is also observed in salivary and mammary glands, dorsal root ganglia, and pancreas [99,110]. FXYD2γ is the γ subunit of the Na^+^/K^+^ ATPase and regulates its activity by inducing ion channel activity by direct binding to the α subunit [109]. By controlling the activity of this ATPase, FXYD2γ is involved in the generation of the resting membrane potential in neurons and Na^+^ reabsorption in kidneys [111]. Hyperosmotic shock triggers the expression of FXYD2γa in proximal tubule cells [112], and FXYD2γa may play a role in the regulation of mechano-sensitivity in rodent cells [113,114]. While the function of FXYD2γa in neurons seems well defined, its role in beta cells remains to be clarified. FXYD2γ knock-out mice display beta cell hyperplasia and glucose intolerance, suggesting that this protein plays a role in endocrine pancreas physiology [110]. FXYD2γa expression is restricted to beta cells and appears early during fetal development [99], which makes it one of the most specific beta cell biomarkers identified so far. To target FXYD2γa for imaging purposes, we generated a short peptide, P88, by using phage display and functionalized it for MRI by conjugation to ultra-small particles of iron oxide (USPIO) [47] or Gd-DOTA [48]. By using these probes, we were able to detect tumors constituted of cells overexpressing the target [47,48], or EndoC-βH1 [48]. Unfortunately, the limited sensitivity of the probe associated with the use of MRI hinders the possibility of detecting BCM and of quantifying their size. Future efforts will be focused on the adaptation of P88 to PET imaging and the generation of a labeled nanobody targeting FXYD2γa.

Besides their potential utility for imaging, the beta cell biomarkers described above may be useful to deliver protective cargo to the beta cells, including the targeting of regulatory T cells (that have the potential to decrease insulitis) to the islets [115]. In another potential application, expression of FXYD2γa in exosomes derived from human beta cells allowed the noninvasive monitoring of rejection of islet allografts [116]. This suggests that FXYD2γa and other specific beta cell biomarkers may allow capturing beta cell-derived vesicles from the serum, with the potential of enabling the following of beta cell destruction via circulating markers. If confirmed, this approach would provide an interesting complement to beta cell imaging and functional tests.

## 6. Other Innovative Approaches for Beta Cell Imaging

Present attempts at BCM quantification by imaging relies on the use of single techniques, such as PET-CT or MRI, and is thus limited to the quantification of only one parameter, the BCM. However, as described above, the function of the remaining beta cells could be hampered by an underlying process such as inflammation and/or severe metabolic stress (“stunned beta cells”) [8]. It would be thus advantageous to use multiple imaging parameters in parallel to asses both beta cell mass and function. Multimodal imaging techniques could enable this goal. A recent study combined the use of two techniques, e.g., MRI and PET imaging [117] based on two different probes, namely manganese (Mn) ions (Mn ions enter beta cells via voltage-dependent calcium channels that are activated in response to glucose metabolism and are thus a potential surrogate for the steps leading to glucose-induced insulin release) and exendin-4 (to quantify BCM) [117]. By this approach, it was shown an increase in both BCM and function in mice that spontaneously develop insulinomas [117]. Despite these promising results, the study had limitations that could hamper its clinical translation. First, exendin-4 and Mn ions accumulation must be quantified at different time points (1 and 24 h, respectively) [117]. Second, clinical use could be limited by the potential neurotoxicity of Mn^2+^ [118,119]. Moreover, data were only generated in mice, and feasibility remains to be demonstrated in humans. Another proposed approach is to detect Zn^2+^ ions accumulation in insulin granules. By using a gadolinium-based Zn^2+^-sensitive agent and MRI it was possible to detect “hot spots” in the abdomen of mice after glucose injection that co-localized with islets as analyzed by histology [53]. The sensitivity of this technique is low, but this approach may become viable with the continuous improvements of MRI instrumentation, and could be used in the future in combination with agents that quantify BCM.

Quantification of insulitis is also an exciting approach to follow up the autoimmune assault in T1D and to estimate the effect of immunosuppressive drugs, particularly if measured in parallel with BCM. Probes used in other fields could be repurposed for this goal. For instance, USPIOs are known to accumulate in macrophages in atherosclerotic lesions [120]. Such magnetic nanoparticles and MRI imaging were used to predict with success the onset of diabetes in NOD mice [121]. In mice, fluorescently labeled paramagnetic beads were used to track and visualize microvascular leakage in NOD mice [122]. Ferumoxtran-based USPIOs were used to detect insulitis in early-onset T1D patients [123], although the signal increase was moderate (2-fold) [123]. Another interesting tool in this context is to use tracers that bind to PDL1 (Programmed death-ligand 1), presently under development for tumor imaging [124]. Pancreatic beta cells exposed to the inflammatory environment of T1D, but not control beta cells, express high amounts of PDL1, probably as an attempt to fend off the autoimmune assault [125]. Thus, imaging PDL1 using non-blocking antibodies could provide an accurate estimation of the islets under immune-mediated attack.

## 7. Could the Beta Cell Imaging Be Deleterious for Beta Cells?

The validation of tracers for BCM imaging must go hand in hand with careful considerations of safety. First, many T1D patients are children, who should not be exposed to excessive radiation. Second, the probes to be used must not present any cytotoxicity against beta cells (i.e., no effect on cell viability, insulin secretion, and DNA integrity), nor stimulate the autoimmune reaction present in type 1 diabetes. Third, the immunogenicity of BCM imaging agents should be low as repetitive imaging sessions will be required to ensure the patient’s follow-up. Fourth, as most of the present techniques rely on the use of PET or SPECT, which require radioisotopes, the radiation level should be kept as low as possible. For instances, concerns were reported about the non-specific uptake of exendin-4 derivatives in the kidneys, and therefore radiation dosimetry. A similar concern could be risen regarding other small-size probes such as Nbs or metabolites that are cleared via the urinary tract. Importantly, independent groups using differently radiolabeled forms of exendin-4 did not detect beta cell or renal toxicity [126,127], and Nbs targeting DPP6, at doses 50-fold higher than the ones present in vivo, did not affect human beta cell survival or function [101]. More recently, a study performed on six patients with hyperinsulinemic hypoglycemia showed that the effective dose of exendin-4 for BCM imaging was 0.71 ± 0.07 mSv (for an injection of 100 MBq) [128]. Since the present maximal annual acceptable radiation exposure for adults in Europe is 20 mSv [129], the technique would be safe for this population. The dose would be however higher in newborns (2.32 ± 0.32 mSv, annual limit = 1 mSv) but decreases rapidly with age (<0.77 mSv in 1-year old children) [128]. Several methods have been suggested to limit kidney probe uptake and radiation exposure, such as pre-administration of lysine, gelofusine, or polyglutamic acid [130], or use of a cleavable linker that can be removed by kidney cells [131,132].

## 8. Radiochemistry—Appending Imaging Isotopes to Beta Cell Probes

Over the last half century, there has been rapid and widespread growth in the use of nuclear imaging driven by the availability of nuclear imaging isotopes and development of novel radiopharmaceuticals, many of them considered for BCM imaging (see above). Nuclear imaging primarily consists of two imaging techniques, PET and SPECT. While there have been efforts to use SPECT in beta-cell imaging [43,45,46,56,57,133], we will focus here on the chemistry of PET isotopes. PET is a nuclear imaging technique used to visualize functional physiological or pathological processes in the body. This is accomplished through the use of unstable isotopes which undergo nuclear β^+^ decay. As part of this nuclear decay process, PET isotopes emit positrons (antimatter counterpart of electrons) which annihilate with electrons located millimeters away, resulting in the emission of two coincident 511 keV gamma photons in opposite directions. A PET scanner measures these gamma photons with respect to time resulting in high spatial resolution of the annihilation event. These annihilation events are reconstructed in silico to produce a three-dimensional image of the tracer concentration within the body over time. When coupled with anatomical imaging techniques, such as CT or MRI, the localization of tracer uptake can be further defined.

Traditionally, the design of radiopharmaceuticals for PET was confined to a few isotopes; carbon-11, nitrogen-13, oxygen-15, and fluorine-18. With improvements in both cyclotron availability, targetry and automated synthesis and purification chemistries, the incorporation of alternative isotopes, especially radiometals, into nuclear imaging probes has drastically increased. With the vast array of radionuclides presently available from both locally sourced cyclotron facilities and commercial distributors of longer-lived isotopes, the choice of radionuclide must work in concert with the chemical form of the unlabeled ligand (small molecule, peptide, antibody, etc). Once the modality and ligand chemistries are known, the radionuclide selection can be narrowed based on other properties such as radioactive half-life (t^1^/_2_), ease of chemical synthesis, and required in vitro and in vivo properties. A wide variety of nuclear imaging isotopes have been employed in tracers geared towards beta cell imaging.

Since carbon is the backbone of all organic molecules essential to life, incorporation of carbon-11 (^11^C, t^1^/_2_ = 20.4 min) is an obvious choice for many PET tracers as the chemistry is straightforward and no changes are made to the parent structure or its pharmacological properties. ^11^C is produced from high-energy proton bombardment of naturally occurring nitrogen-14 on a cyclotron; addition of a small amount of O_2_ gas affords [^11^C]CO_2_ as the primary source for ^11^C chemistries. There are numerous ^11^C synthons that may be derived from [^11^C]CO_2_, many of which can be prepared on commercially available synthesis modules including [^11^C]MeI, [^11^C]MeOTf, [^11^C]CO, and [^11^C]HCN. However, ^11^C has a relatively short half-life, requiring efficient chemistry with incorporation of the ^11^C-label occurring at or near the end of the synthesis to minimize loss of radioactivity. Furthermore, availability is limited to local production facilities as the short half-life restricts the ability to distribute these tracers. Several tracers developed as surrogate markers of beta-cell mass have utilized ^11^C for use with PET including [^11^C]DTBZ [49], [^11^C]5-HTP [50], [^11^C]AZ12204657 [134], and [^11^C]MK7246 [135].

With the widespread clinical use of 2-deoxy-2-[^18^F]fluoro-D-glucose ([^18^F]FDG), fluorine-18 (^18^F, t^1^/_2_ = 109.8 min) is the most frequently used PET nuclide. ^18^F is typically prepared on a cyclotron through proton bombardment of oxygen-18 enriched water. The aqueous fluoride solution from the cyclotron is formulated with aqueous potassium carbonate and the crown ether Kryptofix^®^ 222 to chelate the potassium counter-ion and enhance the reactivity of the fluoride ion. The chemistries to incorporate ^18^F have been extensively reviewed [136,137,138,139]. The most common radiofluorination technique is nucleophilic substitution of halides or sulfonates by the [^18^F]-fluoride ion. Since fluoride is not a strong nucleophile in aqueous solution, the [^18^F]-fluoride is dehydrated prior to the reaction to further enhance reactivity. The longer half-life of ^18^F relative to ^11^C allows for more complex, multi-step syntheses, extended imaging times, and distribution of tracers beyond the site of preparation. Furthermore, the prevalence of fluorinated pharmaceuticals has drastically increased in recent years as the substitution of hydrogen or a hydroxyl group for fluorine in biologically active small molecules is generally well tolerated. Thus, ^18^F can be incorporated into small molecules with little to no change in biological activity. Several ^18^F-tracers have been evaluated for their ability to quantify beta cell mass including ^18^F-exendin analogs [140], 5-(2-[^18^F]-fluoroethoxy)-L-tryptophan [141], [^18^F]fluoropropyl-(+)-DTBZ [71], ^18^F-mitiglinide derivatives [142], and ^18^F-repaglinide derivatives [143].

The radiometal gallium-68 (^68^Ga) PET has garnered much attention in recent years, particularly following FDA approval of two ^68^Ga agents (^68^Ga-DOTATATE (DOTA-0-Tyr3-Octreotate) and ^68^Ga-DOTATOC (DOTA^0^-Phe^1^-Tyr^3^)octreotide)) and the success of ^68^Ga-agents targeting prostate-specific membrane antigen (PSMA) in prostate cancer clinical studies. Despite a relatively short half-life of 68 min, ^68^Ga tracers are readily deployed as preparation of the ^68^Ga isotope is not dependent on a cyclotron. Commercial gallium-68 generators extract ^68^Ga from a source of decaying germanium-68 (^68^Ge, t ^1^/_2_ = 271 days). Given the inherent finite lifetime of gallium generators, solutions have been developed to prepare ^68^Ga on demand from a cyclotron via proton bombardment of an aqueous solution of zinc-68 [144]. Unlike carbon-11 and fluorine-18, ^68^Ga-labeling requires the use of a chelator to attach the radiometal to the desired ligand, as described in excellent reviews detailing the coordination chemistry and the application of specific chelators [145,146,147]. The most common ^68^Ga-chelators include the macrocycles DOTA and 1,4,7-triazacyclononane-1,4,7-triacetic acid (NOTA). Once the conjugation of the chelator to the ligand is complete, the complex is combined with an aqueous solution of ^68^Ga to capture the radiometal. By conjugating the chelator prior to the introduction of the radiometal, the labeling process is significantly shortened. This also allows for the use of a broad range of imaging agents, including small molecules, peptides, and antibody fragments. Numerous tracers incorporating ^68^Ga to image beta-cell mass have been reported, including exendin-4 derivatives [148,149], octreotide derivatives [150], and DPP6-targeting nanobodies [101].

An emerging application of PET imaging, dubbed immuno-PET, uses radiolabeled mAbs as the diagnostic targeting vector. With the optimization of cyclotron targetry and methodology, zirconium-89 (^89^Zr) has emerged as an optimal isotope for immuno-PET imaging. ^89^Zr is typically produced from high-energy proton bombardment of an Yttrium-89 solid target (Yttrium foil over aluminum/copper disc). The decay half-life of ^89^Zr (3.3 days) matches the typical in vivo pharmacokinetics of mAbs to give an optimal signal to background ratios. Similar to other radiometals, ^89^Zr labeling of antibodies requires the use of a chelator chemically linked to the antibody, with desferrioxamine B (DFO-B) being the most common chelator for ^89^Zr labeling. The long half-life of ^89^Zr also allows for the broad commercial distribution of the isotope. The increasing availability of ^89^Zr has led to more clinical studies of ^89^Zr tracers with promising results. While the number of ^89^Zr tracers specifically targeting beta cells is limited, there have been successful feasibility studies utilizing ^89^Zr-labeled mAbs targeting transmembrane protein 27 [151].

While imaging with ^89^Zr-mAbs can provide outstanding affinity and target specificity, the long half-life of ^89^Zr could be deleterious to beta cells due to prolonged radiation exposure. To mitigate the prolonged exposure to radiation from ^89^Zr, pretargeting approaches may be used. Pretargeting takes advantage of biorthogonal click chemistry in vivo between trans-cyclooctenes conjugated to mAbs and tetrazine-based small molecules [152,153,154]. In this approach, the modified antibody is administered, followed by a delay period of 24–72 h to allow time for the antibody to circulate and engage the desired target. The radiolabeled tetrazine-containing small molecule probe that recognizes and specifically binds to the transcyclooctenes of the modified antibody is then administered. This system combines the favorable pharmacokinetic properties of small molecule tracers with the affinity and specificity of antibodies. As such, short-lived isotopes such as ^18^F, ^64^Cu, and ^68^Ga, which are normally not compatible with antibody imaging due to the mismatch of the short half-life of the isotope and the long in vivo circulation times of the mAb, are now viable alternatives to ^89^Zr for mAb imaging. This approach could prove to be extremely valuable for beta cell imaging as a method to obtain specificity while simultaneously reducing radiation exposure to the beta cells.

## 9. From Preclinical Validation to First in Man Testing—Checking all the Required Steps

Conventional pharmaceuticals are a class of compounds designed to bind selectively and with high affinity to an epitope of interest in the target tissue, with the intent of altering the disease-relevant biological processes while minimizing the off-target (i.e., non-specific) binding and associated toxicity. When combined with appropriate dose amount and frequency, the result is a drug, which has excellent target tissue exposure, minimal metabolism, and low off-target toxicity. Therefore, the primary goal of developing conventional pharmaceuticals is the efficacy and safety profile of the drug. By contrast, molecular imaging probes represent a special class of pharmaceuticals, which retain the selectivity and affinity of traditional pharmaceuticals but because they are often labeled with radioisotopes [155], require dose levels that are far below the amount required to elicit either a pharmacological or toxicological response [155], and typically operate at levels significantly below the No Effect Level (NOEL) [155,156]. This microdosing approach is only possible if the specific activity (i.e., the activity per quantity of a radionuclide) is sufficiently high to permit non-invasive detection while minimizing the on-target effect of the unlabeled probes mass [157,158]. As such, the primary objective of the imaging probe is the non-invasive detection of the physiological process, which underlies the disease progression (i.e., pathophysiology), and/or therapeutic response, which modifies the disease trajectory (i.e., pharmacodynamic biomarker), without altering the state of this system. To achieve this, molecular imaging probes should possess the following characteristics:

- High target binding affinity. This characteristic is among the most important attributes of an imaging probe since the binding of the agent to the epitope of interest is one of the key features that promote tissue accumulation. To achieve high tissue uptake within the mean resonance time of the tracer in the primary circulation, the tissue binding must exhibit fast on- ($k_{on}$) and slow off- ($k_{off}$) rate constants for the target epitope.
- High target specificity. Target specificity ensures that the molecular probe is reporting only the target of interest and the underlying physiological processes, but also plays a role in improving the signal-to-noise ratio (SNR) of the resulting image. Low specificity agents have high off-target (i.e., non-specific) binding, thus increasing the apparent “noise” floor of the image, which is unrelated to the physiological processes, thus making visualization and quantification of the image more difficult.
- High target sensitivity. In order to detect the pathophysiology of the disease process at the early stages, where the epitope of interest may be at low concentration, the molecular probe should have high sensitivity. In other words, trace quantities of the probe must be able to bind to the target with high affinity and specificity (see above), such that they produce an image that can differentiate the underlying pathology from normal physiological function. Implicit in this is that the molecular probe must function at trace concentrations, such that it does not elicit a pharmacological response (i.e., administered in trace quantities) and is below the toxicological NOEL.
- High in vivo stability. Despite the fact that only trace amounts of the imaging probe are administered to the subject, it is critical that the structure remains intact since numerous enzymes and proteases are present in the blood, liver, and target tissue. If a compound undergoes transformation, then the three-dimensional structure of the probe will change, thus affecting the binding of the compound to the pocket and epitope of interest. Moreover, if a compound undergoes cleavage because of its metabolism, and the dynamics of this new metabolite are ignored, or not accounted for, the interpretation of the subsequent images are significantly biased by the presence of the new metabolite(s).
- High contrast to noise ratio. Image contrast is also a key factor to consider when developing a molecular imaging probe. To achieve this, images typically have high SNR and low off-target binding (i.e., low non-specific), which yields target-to-background ratios suitable for the detection of trace quantities in the target tissues. Moreover, imaging probes should have kinetics that favors rapid uptake and slow washout rates in the target tissue, while non-target tissues show preferentially fast washout rates. This differential rate is in part related to the wash-in kinetics, affinity of the ligand to the target, and low non-specific binding to non-target tissues.
- Low toxicity and immunogenicity. Since imaging probes are a special class of pharmaceutical administered in trace quantities, toxicity is rarely an issue. However, if an imaging probe is composed of a labeled biologic, e.g., antibodies or nanobodies, immunogenicity can present a unique set of challenges, since repeat administration could result not only in an immune response, thus rendering the targeting inactive, but also lead to severe allergic reactions.
- Low production costs. The cost of imaging probes production is governed in part by the cost of the starting materials, cost of cyclotron beam time, and labor associated with reaction series. Of particular importance pre-clinically is the selection of the nuclide: the longer the half-life, the greater numbers of products that can be derived from a production run, and the broader the distribution range of the formulated drug products.

In an effort to evaluate if a novel imaging agent retains the above-listed characteristics, the majority of molecular imaging probes undergo rigorous in vitro and in vivo testing to determine their physiochemical, kinetic, and biological properties. In the in vitro phase, there are several overlapping, interactive and recursive stages in the evaluation of imaging probes, which include the establishment of assay conditions, validation of the assay, testing novel ligands in the assay, and quantitative analysis of the resulting data to derive binding parameters [159].

Recent advances in assay miniaturization and the use of 96, 384, or 1536 well plates permits the high throughput screening (HTS) of large numbers of molecular probes in a single plate [160]. In these assays, the concentration of receptors per unit volume is maximized, while the concentration of radioligand is kept to a minimum to reduce costs and increase assay sensitivity [160]. When using high-affinity molecular ligands, there is a risk that the binding of ligand to the receptor will result in a reduction in the free ligand concentration (i.e., ligand depletion), thus violating the assumption that free ligand concentration is equal to the concentration added [160]. Instead, the free ligand concentration should be measured directly, where the concentration of receptors should be less than 10% of the ligand’s equilibrium dissociation constant (Kd), and thus ideally, less than 10% of the ligand will be bound to the receptor of interest [161]. Importantly, these assay conditions are often incompatible with miniaturization formats, resulting in a conflict between precision and practicality. An alternative approach is to take radioligand depletion into account [162,163,164]; however, it is suggested that these analyses should only be used in assays with less than 50% ligand depletion [163].

Once the relationship between the ligand and the concentration of the receptor has been established, the determination of the molecular probe affinity may be performed. Under standard conditions, receptor binding reactions follows the law of mass action according to:

(1) L+R↔L·R

At equilibrium, the ligands dissociation rate constant can be described as follows

(2) $$K_{d}=\frac{k_{off}}{k_{on}}=\frac{\left[L\right]*\left[R\right]*k_{off}}{\left[L\cdot R\right]*k_{on}}$$

where, [L], [R], [L·R], $k_{off}$, $k_{on}$, and $K_{d}$ are the concentration of the ligand, the concentration of the receptor, concentration of the ligand/receptor complex, dissociation rate constant, association rate constant, and equilibrium dissociation constant, respectively [165]. Importantly, molecular probes showing binding to the target site, which are typically limited in their quantity and distribution [166,167,168], are, therefore “specific” and saturable at increasing concentrations of the radioligand, while probes which bind to off-target sites are “non-specific” and have virtually unlimited binding sites [159,162,165,169]. Using this construct, several methods have been developed for the determination of the affinity of a ligand for its target.

The most direct approach is the application of saturation binding, where the total binding is determined as a function of the concentration of the ligand of interest [164,166,167]. To accurately determine the affinity, the radiotracer concentrations should range from 10-fold below to 10-fold above the expected Kd [166]. Moreover, to determine the degree of non-specific binding, studies have shown that if the assay is conducted at 100–1000 fold above the Kd (i.e., excess cold/unlabeled), the binding curve is linearly dependent of dose [164,165]. Provided this, the specific binding can be determined by subtracting the non-specific amount, and then analytically modeling the curves. In early studies, this modeling was performed by linearizing the data by plotting the bound versus bound/free and fitting the equation of a line through the data, where the slope of the line is equal to −1/Kd, and the intercept with the *X*-axis is an estimate of maximum density of binding sites (Bmax) [170]. Although this approach is useful for visualizing data, it suffers from inaccuracies that violate the assumptions of linear regression, thus impacting the Kd and Bmax estimates [165,168]. Modern approaches utilize direct modeling via non-linear regression [165], where the data are plotted as the concentration of the ligand versus binding (in disintegrations per minute, DPM), and are modeled according to the following

(3) $$Y=\;\frac{B_{max}*\left[L\right]}{K_{d}+\left[L\right]}$$

where $B_{max}$, $K_{d}$, and [L] are the maximum density of binding sites, the equilibrium dissociation rate constant, and the concentration of the ligand. Unlike the linearization, this approach has been shown to correctly estimate the rate constants without introducing bias and does not violate the primary assumptions of dependency and normal distribution of the data [159,165].

Under conditions where the supply of radioligand is limited, estimates of affinity can be determined via competitive binding, where a single concentration of a radiolabeled ligand is used in the presence of various concentrations of unlabeled ligand, where the logarithm of the unlabeled ligand is plotted against the total ligand binding. Under these conditions, the estimate of total binding can be determined via non-linear regression of the following

(4) $$Y=\;NS+\frac{\left(T-NS\right)}{1+10^{\left(\left[L\right]-\log10\left(IC_{50}\right)\right)}}$$

where NS, T, [L], log10, and $IC_{50}$ are the nonspecific binding, total binding, concentration of unlabeled ligand, logarithm base 10, and inhibitory concentration at 50%, respectively. Provided this, and if the Kd of the labeled is known, equilibrium dissociation constant for inhibitory studies (Ki) can be computed based on Chang-Prusoff transformation [171].

In order to understand the association (i.e., on-rate) and dissociation (i.e., off-rate) of the labeled molecular probe to the receptor system, kinetics studies with time can be performed independently for each, or combined as a single study [165]. Under conditions of a single combined study, the receptor system is loaded with radioligand, and periodic measurements are made with time until equilibrium has been achieved [164,165]. At equilibrium, the receptor system is centrifuged, re-suspended in fresh buffer, and 100–1000 fold unlabeled ligand is added. This is followed by a detailed analysis of the assay until the new equilibrium has been achieved [164,165]. From this time course, the association and disassociation rate constants can be modeled as follows

(5) $$Y=\;\{\begin{array}{c} t<t_{n};\;\frac{B_{max}*\left[L\right]}{\left(\left[L\right]+\frac{k_{off}}{k_{on}}\right)}*\left(1-e^{\left(-1*\left[L\right]*k_{on}+k_{off}*t\right)}\right)\\ t\geq t_{n};\;\frac{B_{max}*\left[L\right]}{\left(\left[L\right]+\frac{k_{off}}{k_{on}}\right)}*\left(1-e^{\left(-1*\left[L\right]*k_{on}+k_{off}*t_{n}\right)}\right)*e^{\left(-1*\left[L\right]*k_{off}\left(t-t_{n}\right)\right)} \end{array}$$

where t, $t_{n}$, $B_{max}$, [L], $k_{on}$, and $k_{off}$ are the time, equilibrium time when the excess unlabeled is added, the maximum density of binding sites, labeled ligand, association (“on”) rate constant, and dissociation (“off”) rate constant, respectively. From these rate constants, estimates of the Kd ($\frac{k_{off}}{k_{on}}$) [164,165] and binding potential (Bp; $\frac{k_{on}}{k_{off}}$) [172] can be derived. Molecular probes that have high affinity and the likelihood of advancing to in vivo studies typically have a Kd of <50 nM [159,165], specific binding (i.e., specific/non-specific of >5 fold) and a Bp > 0.02. Recent work from our lab has combined the traditional association-dissociation studies this with miniaturization and autoradiography to permit a single plate determination of $k_{on}$ and $k_{off}$ rates [173].

**Figure 3:**
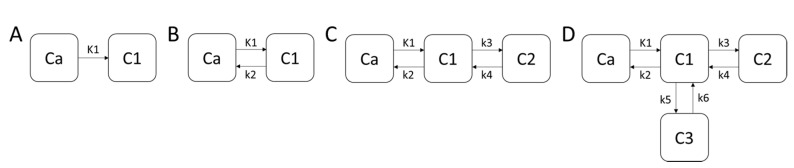
The block diagrams (**A**) is the most simplistic model achievable and has been used to describe trappable tracers for blood flow determination such as microspheres [192] (Equation (6)), while (**B**) represents a similar model, but the tracer is not trapped in the C1 compartment, thus allowing efflux back into the blood as with radioactive water studies [178] (Equation (7)). In diagram (**C**), the model has been used to describe the binding of ligands to receptor systems, where the C1 compartment represents the interstitial space, and C2 is the receptor complex on the tissue of interest, such as for glucose metabolism [193] or P2X7 receptor binding [173] (Equations (8) and (9)). Lastly, (**D**) has been used to describe binding to the receptor system described in (**C**), but the C3 compartment represents the non-specific binding (Equations (10)–(12)) [194].

Once a molecular probe has been well characterized via the aforementioned in vitro assessments and has been shown to exhibit high affinity (i.e., <50 nM), favorable association and dissociation kinetics (i.e., $k_{on}\gg k_{off}$), in vivo studies, can be performed in model systems, such as mice implanted with human pancreatic islets. These studies typically encompass selecting an animal model which exhibits the biology of interest (e.g., tracers targeting splice variants are often species-specific and cannot be evaluated in mouse tissues), and therefore the receptor system to image, and performing medical imaging of the radiotracer via PET (or SPECT). Most molecular probes are neither covalently bound to their target nor are functionally trapped in the tissues [174,175]. Thus, in order to understand the kinetics of the radiotracer, studies must be performed via dynamic acquisition, where three- dimensional (3D) images with time (i.e., four-dimensional image series) are acquired to permit non-invasive monitoring of the molecular probe. In this acquisition scheme, vascular access is acquired prior to the subject being placed on the scanning bed and advanced to the center of the scanner’s field of view (FOV). After collection of attenuation correction scans via computed tomography (CT), the PET (or SPECT) scanner acquisition sequences start to permit several seconds of data acquisition prior to tracer administration and are then continued for 45–60 min. Starting the scanner prior to administration of the molecular probe permits the collection of images during the arterial phase of the tracer kinetics and is critical for subsequent tracer kinetic modeling. At the completion of the acquisition phase, 3D image series are reconstructed into discrete time bins with a reverse logarithmic spacing (i.e., 0, 1, 2, 3, 4, 8, 16, 24, 36, 48, 60 min), yielding a series of image volumes suitable to capturing the appearance of the tracer in the arterial tree with time that tracks the bio-distribution of the radioligand as it moves through the subject [176]. To capture a time course, images are segmented across time for a major arterial vessel and the tissue(s) of interest, and when combined with the kinetic tracer model, the theoretical distribution of the tracer over the scanning period can be quantified [176]. In this system, the mathematical model defines the relationship between the measurable imaging data and relates this to the physiological parameters that affect the uptake and metabolism of the molecular tracer following the conservation of mass [176,177]. A number of literature sources provide a comprehensive presentation of modeling alternatives [178,179,180,181,182,183], where some are described as stochastic or non-compartmental and require minimal assumptions concerning the underlying physiology of the tracer’s uptake and metabolism [184]. Alternatively, a class of models that specify the physical locations, biochemical forms of the tracer, and also include the concentration gradients that exist, have been developed to describe the capillary–tissue exchange of a tracer and are termed distributed [185,186,187,188,189,190,191]. A class of model that is between a stochastic and distributed model is the compartmental model, that attempts to retain underlying physiology but do not include concentration gradients [176,177]. In PET (or SPECT), a compartmental model utilizes the measurements of radioactivity in a specific organ, region, or even pixel collected via the scanner with time. If the tracer enters and exits the organ via the blood, then the tracer kinetics in other body regions need not be considered to evaluate the physiological traits of the organ of interest [176,177], thus allowing each region or pixel to be analyzed independently. The type of tracer kinetic model to select and the relationship to the underlying physiology is beyond the scope of this review, and the reader is referred to excellent literature sources on this topic [176,177]. Regardless of the model selected, the end product is the generation of rate constants that describe the flux of radioactivity in and out of theoretical compartments of interest. Below are block diagrams for the 4 most common models used in tracer kinetic modelling (Figure 3):

Given the models described in Figure 3, the following partial differential equations can be formulated to describe the conservation of mass of the radiotracer between compartments.

**Model A**

(6) $$\frac{dC1}{dt}=K1C1$$

**Model B**

(7) $$\frac{dC1}{dt}=K1C1-k2C1$$

**Model C**

(8) $$\frac{dC1\left(t\right)}{dt}=K1*C1\left(t\right)-k2*C1\left(t\right)$$

(9) $$\frac{dC2\left(t\right)}{dt}=k3*C1\left(t\right)-k4*C2\left(t\right)$$

**Model D**

(10) $$\frac{dC1\left(t\right)}{dt}=K1*C1\left(t\right)-k2*C1\left(t\right)$$

(11) $$\frac{dC2\left(t\right)}{dt}=k3*C1\left(t\right)-k4*C2\left(t\right)$$

(12) $$\frac{dC3\left(t\right)}{dt}=k5*C1\left(t\right)-k6*C3\left(t\right)$$

where, Equations (6)–(12), correspond to the block diagrams A, B, C, and D shown in Figure 3, respectively. Typically, these equations are solved numerically, to yield the rate constants K1–k6, and can be reformulated to yield physiological information as follows:

(13) $$\dot{Q}=K1$$

(14) $$Kd=\frac{k3}{k4}=\frac{k_{off}}{k_{on}}$$

(15) $$Bp=\frac{k4}{k3}=\frac{k_{on}}{k_{off}}$$

(16) $$NS=\frac{k5}{k6}$$

(17) $$Vt=\frac{K1}{k2}\left(1+\frac{k3}{k4}\right)$$

where, $\dot{Q}$, Kd, Bp, NS, and Vt are the tissue perfusion, equilibrium dissociation rate constant, equilibrium binding potential, non-specific binding, and total volume of distribution of the receptors in the tissues. These methods provide the only non-invasive means to determine these parameters for the receptors in vivo, and are the fundamental basis for target specificity, target sensitivity, and high contrast to noise, and are necessary for understanding the pathophysiological trajectory of disease. Moreover, when combined with therapeutics, which directly modulate the receptor system, the molecular probe provides pharmacodynamic readouts of target engagement and therapeutic responses.

The administration of any compound, which is either experimental or for clinical trials, requires extensive information to be generated in preparation for human administration to ensure a complete understanding of the benefit to risk ratio profile [195]. In order to obtain these data, preclinical studies are performed in model systems in vivo and/or in vitro, where a more complete understanding of the compounds kinetics, distribution, and toxicology are measured [195,196]. In the case of a radiopharmaceutical, the kinetics of the tracer are obtained by the aforementioned dynamic study, where the tracer kinetics are derived from the image time series. The biodistribution, by contrast, may be obtained by either post-mortem determination of the tissue accumulation or via image-based quantitation. In traditional post-mortem technique, either at the completion of a PET (or SPECT) scan, or in a dedicated cohort which has been injected with the radiotracer, major organs (i.e., brain, heart, lungs, liver, kidneys, spleen, pancreas, small and large intestines, bladder, and skeletal muscle) from the test subject are extracted, weighed, and total activity measured to determine the amount of radiopharmaceutical which is present per gram of tissue [195]. Similarly, recent work with theranostic agents has utilized image-based distribution approaches, where regions of interest are drawn on PET images, guided by co-registered anatomical CT or MRI images [196], and yield similar endpoints to the post-mortem analyses conducted ex vivo. Regardless of the approach, the end goal in each of these cases is the determination of tissue radiopharmaceutical exposure, and therefore the radiation dosimetry of the molecular probe. Radiation dosimetry quantifies the amount of ionizing radiation deposited in a tissue, and is influenced by the energy, size, charge, half-life, administered dose, and the dose rate of the radioactive material [197,198], and is used to determine the relative risk to the subject. To determine this, data collected from preclinical biodistribution studies can be scaled allometrically, and dosimetry estimates extrapolated to humans using tools such as IDAC [199], OLINDA [200], and MIM SurPlan [201]. Independent of the approach, the end goal is the determination of the tissue dosimetry estimates to help guide the use of the new radiopharmaceutical by researchers and clinicians alike.

## 10. Conclusions

There are presently no fully adequate ways to quantify beta cell mass in vivo or to target protective agents specifically to the beta cells; this hampers the understanding of the pathogenesis of diabetes and the development of novel therapies to preserve beta cells and/or to induce their proliferation. To solve this problem, novel and specific beta cell biomarkers must be identified to allow adequate in vivo imaging by methods such as PET and, in future steps, specific delivery of therapeutic agents. The ideal biomarker should enable the development of tracers for measurements based on minimally invasive technology, allowing repeated examinations over time. The eventual success of beta cell imaging will allow the stratification of patients at early diagnosis (i.e., define the amount of remaining beta cells and follow them up as the disease evolves) and, crucially, to assess and validate novel therapies aiming to preserve and/or restore beta cell mass, including islet transplantation, immune modulation and new agents that induce the generation of novel beta cells. We are not there yet, but the presently discussed recent advances in the field, allied to logic and a step-by-step approach to identify and validate novel tracers for beta cell imaging, augurs well for the future of the field.

## Abbreviations

3D	Three-dimensional
BCM	Beta cell mass
DOTA	Dodecane tetraacetic acid
DOTATATE	DOTA-^0^-Tyr^3^-octreotate
DOTATOC	(DOTA^0^-Phe^1^-Tyr^3^) octreotide
DPP6	Dipeptidyl peptidase 6
DTBZ	Dihydrotetrabenazine
DTPA	Diethylenetriaminepenta-acetic acid
FOV	Field of view
FXYD2	FXYD domain-containing ion transport regulator 2
GLP-1	Glucagon-like peptide-1
GLP-1R	Glucagon-like peptide-1 receptor
HTP	Hydroxytryptophan
HTS	High throughput screening
IEQ	Islet equivalent
mAb	Monoclonal antibody
MRI	Magnetic resonance imaging
Nb	Nanobody
NOD-SCID	Nonobese diabetic/severe combined immunodeficiency
NOEL	No effect level
NOTA	1,4,7-triazacyclononane-1,4,7-triacetic acid
PET	Positron emission tomography
PD-L1	Programmed death-ligand 1
PSMA	Prostate-specific membrane antigen
SNR	Signal-to-noise ratio
SPECT	Single-photon emission computed tomography
T1D	Type 1 diabetes
T2D	Type 2 diabetes
USPIO	Ultra-small particles of iron oxide
VMAT2	Vesicular monoamine transporter 2
